# Muscle NAD^+^ depletion and Serpina3n as molecular determinants of murine cancer cachexia—the effects of blocking myostatin and activins

**DOI:** 10.1016/j.molmet.2020.101046

**Published:** 2020-06-26

**Authors:** J.J. Hulmi, F. Penna, N. Pöllänen, T.A. Nissinen, J. Hentilä, L. Euro, J.H. Lautaoja, R. Ballarò, R. Soliymani, M. Baumann, O. Ritvos, E. Pirinen, M. Lalowski

**Affiliations:** 1 Faculty of Sport and Health Sciences, NeuroMuscular Research Center, University of Jyväskylä, Jyväskylä, Finland; 2 Department of Physiology, Faculty of Medicine, University of Helsinki, Helsinki, Finland; 3 Department of Clinical and Biological Sciences, University of Turin, Turin, Italy; 4 Research Program for Clinical and Molecular Metabolism, Faculty of Medicine, University of Helsinki, Helsinki, Finland; 5 Stem Cells and Metabolism Research Program, Faculty of Medicine, University of Helsinki, Helsinki, Finland; 6 Meilahti Clinical Proteomics Core Facility, HiLIFE, Faculty of Medicine, Biochemistry and Developmental biology, University of Helsinki, Helsinki, Finland

**Keywords:** C26, Cancer cachexia, Activin receptor, Nrk2, APR, OXPHOS

## Abstract

**Objective:**

Cancer cachexia and muscle loss are associated with increased morbidity and mortality. In preclinical animal models, blocking activin receptor (ACVR) ligands has improved survival and prevented muscle wasting in cancer cachexia without an effect on tumour growth. However, the underlying mechanisms are poorly understood. This study aimed to identify cancer cachexia and soluble ACVR (sACVR) administration-evoked changes in muscle proteome.

**Methods:**

Healthy and C26 tumour-bearing (TB) mice were treated with recombinant sACVR. The sACVR or PBS control were administered either prior to the tumour formation or by continued administration before and after tumour formation. Muscles were analysed by quantitative proteomics with further examination of mitochondria and nicotinamide adenine dinucleotide (NAD^+^) metabolism. To complement the first prophylactic experiment, sACVR (or PBS) was injected as a treatment after tumour cell inoculation.

**Results:**

Muscle proteomics in TB cachectic mice revealed downregulated signatures for mitochondrial oxidative phosphorylation (OXPHOS) and increased acute phase response (APR). These were accompanied by muscle NAD^+^ deficiency, alterations in NAD^+^ biosynthesis including downregulation of nicotinamide riboside kinase 2 (*Nrk2*), and decreased muscle protein synthesis. The disturbances in NAD^+^ metabolism and protein synthesis were rescued by treatment with sACVR. Across the whole proteome and APR, in particular, Serpina3n represented the most upregulated protein and the strongest predictor of cachexia. However, the increase in Serpina3n expression was associated with increased inflammation rather than decreased muscle mass and/or protein synthesis.

**Conclusions:**

We present evidence implicating disturbed muscle mitochondrial OXPHOS proteome and NAD^+^ homeostasis in experimental cancer cachexia. Treatment of TB mice with a blocker of activin receptor ligands restores depleted muscle NAD^+^ and *Nrk2*, as well as decreased muscle protein synthesis. These results indicate putative new treatment therapies for cachexia and that although acute phase protein Serpina3n may serve as a predictor of cachexia, it more likely reflects a condition of elevated inflammation.

## Highlights

• Cachectic muscle proteome shows decreased OXPHOS and increased acute phase response.
• Cancer cachexia is characterized by lowered muscle *Nrk2* expression and NAD^+^ levels.
• Blocking activin receptor 2B ligands rescues muscle NAD^+^ homeostasis in cachexia.
• Blocking activin receptor 2B ligands prevents affected protein synthesis in cachexia.
• Serpina3n predicts cachexia and cancer-induced APR independently from muscle atrophy.

## Abbreviations

36B4: acidic ribosomal phosphoprotein P0
ACC: Acetyl-CoA Carboxylase
ACVR2B-Fc: activin receptor type IIB
AMPK: adenosine monophosphate activated protein kinase
ANOVA: analysis of variance
APP: acute phase protein
APR: acute phase response
BCA: bicinchoninic acid
B-H: Benjamini-Hochberg correction
BM: body mass
C26: colon-26 carcinoma
COX: cytochrome c oxidase
CTRL: control mice
DEP: differentially expressed protein
FDR: false discovery rate
GAPDH: glyceraldehyde 3-phosphate dehydrogenase
GDF11: growth and differentiation factor 11
DIA nano-LC-HDMS^E^: data independent acquisition nano-liquid chromatography high definition tandem mass spectrometry
DMEM: Dulbecco's Modified Eagle's Medium
FBS: fetal bovine serum
GA: Gastrocnemius
HPRT: hypoxanthine phosphoribosyltransferase 1
PBS: phosphate buffered saline
IgG: immunoglobulin G
IL-6: interleukin 6
IL-15: interleukin 15
INSR: insulin receptor
i.p.: intraperitoneal
MCP-1: monocyte chemoattractant protein-1
mTORC: mechanistic target of rapamycin complex
NAD^+^: nicotinamide adenine dinucleotide
NAMPT: nicotinamide phosphoribosyl transferase
NAPRT: nicotinate phosphoribosyltransferase
NMNAT: nicotinamide mononucleotide adenylyl transferase
OXPHOS: oxidative phosphorylation
OXPHOSome: OXPHOS proteome
PARP: poly(ADP-ribose) polymerase
PCA: Principal Component Analysis
PGC-1α: peroxisome proliferator-activated receptor gamma coactivator 1 alpha
PPARA: peroxisome proliferator-activated receptor-alpha
PPARGC1A and PPARGC1B: peroxisome proliferator-activated receptor gamma coactivator 1 alpha and beta
PVDF: polyvinylidene difluoride
QPRT: quinolinate phosphoribosyltransferase
ROS: reactive oxygen species
Rn18s: 18S ribosomal RNA
RT-PCR: quantitative reverse transcription polymerase chain reaction
sACVR: soluble ligand binding domain of activin receptor type IIB
sACVR/b: sACVR administration before tumour formation
sACVR/c: continued sACVR administration before and after tumour
SDH: succinate dehydrogenase
SDS-PAGE: sodium dodecyl sulphate polyacrylamide gel electrophoresis
SEM: standard error of the mean
Serpina3n: serpin family A member 3n
SIRT1: sirtuin-1
STAT3: signal transducer and activator of transcription 3
SUnSET: surface sensing of translation
TA: tibialis anterior
TB: tumour-bearing
TBP: TATA-binding protein
TCA cycle: tricarboxylic acid cycle
TDO: tryptophan 2,3-dioxygenase
TGF-β: transforming growth factor-β

## 1 Introduction

Cancer cachexia is a multiorgan syndrome characterised by loss of body mass (BM) due to muscle and adipose tissue wasting in the majority of cancer patients [1]. Tumour-derived factors and systemic inflammation are considered the main drivers of cachexia [1]. Inflammation leads to an increase in the concentration of cytokines and is associated with dysregulated metabolism, including mitochondrial dysfunction, which can further contribute to muscle loss [2]. Muscle loss in cancer cachexia is strongly linked with increased morbidity and mortality [3]. Moreover, in preclinical animal models, prevention of cancer cachexia improved survival without effects on tumour growth [[4], [5], [6]], suggesting possible causality between muscle loss prevention and improved survival. However, the underlying mechanisms are unknown.

Proteins from the transforming growth factor-β (TGF-β) superfamily are cytokines, which among others are upregulated in diseases such as cancer. For instance, the expression of TGF-β superfamily proteins, activins A and B, is increased in many human [7] and murine cancer cell lines and tumours [4]. Blood activin A is also elevated in patients with cancer cachexia [8]. Activins A and B and other TGF-β family members such as myostatin and growth and differentiation factor 11 (GDF11) are negative regulators of muscle mass [[9], [10], [11]]; they can be blocked using various strategies such as administration of soluble ligand binding domain of their receptor, namely, activin receptor type IIB (sACVR2B-Fc, herein referred to as sACVR), which effectively increases muscle mass in mice [4,[12], [13], [14], [15]], and humans [16]. Blocking ACVR ligands prolongs survival and reverses cancer cachexia in mice [[4], [5], [6]]. We previously demonstrated improved survival in Colon-26 carcinoma (C26) TB-mice with continued sACVR administration after tumour formation but not when it was administered only before cancer engraftment to increase muscle mass before cachexia [4]. The administration of sACVR attenuated muscle loss in limb and respiratory muscles [4] and improved antioxidant protection in muscle tissue of TB-mice [17], without alterations in tumour growth and inflammation. However, blocking ACVR-signalling did not show major effects at either the metabolome [18] or microbiome level [19]. Thus, no explanation has been provided for the improved survival in sACVR-treated mice.

We applied 3 protocols to compare the impact of blocking ACVR2 ligands: i) administration before the tumour formation to increase muscle size only before the onset of cachexia, ii) administration both before and after the onset of cachexia, or iii) treatment after the tumour is present. These comparisons were made to further understand, at the muscle level, why improved survival was achieved when anti-cachexia treatment was continued after tumour formation but not when it was administered only as a preventive measure [4]. We first conducted muscle proteomic profiling from mice bearing the C26 cancer with or without administration of sACVR. Thereafter, we functionally validated our results to reveal Serpina3n as a predictor of cachexia, but not a generic marker of alterations in muscle mass, and discovered altered nicotinamide adenine dinucleotide (NAD)^+^ metabolism in cancer, which was rescued upon treatment with sACVR.

## 2 Materials and methods

### 2.1 Mice

Male BALB/c (BALB/cAnNCrl) mice aged 5–6 weeks (Charles River Laboratories) were housed under standard conditions (temperature 22 °C, 12:12 h light/dark cycle) with free access to food pellets (R36; 4% fat, 55.7% carbohydrate, 18.5% protein, 3 kcal/g, Labfor, Stockholm Sweden) and water.

The treatment of animals was in strict accordance with the European Convention for the protection of vertebrate animals used for experimental and other scientific purposes. The protocols were approved by the National Animal Experiment Board, and experiments were conducted in accordance with the guidelines of the committee (permit number: ESAVI/10137/04.10.07/2014).

### 2.2 Tumour cell culture

C26 cells were maintained in complete Dulbecco's Modified Eagle's Medium (DMEM, high glucose, GlutaMAX™ supplement, pyruvate, Gibco™, Life Technologies) supplemented with penicillin (100 U/mL), streptomycin (100 μg/mL) and 10% FBS.

### 2.3 Experimental design

Experiment 1: mice were randomised into 1 of 4 body-weight matched groups as described earlier [4]: 1) healthy control mice (CTRL) were injected with PBS vehicle, 2) C26 TB-mice received vehicle administration throughout the experiment (C26 + PBS), 3) C26 TB-mice received only prophylactic sACVR administration before tumour formation (until day 1 after C26 cell inoculation), followed by vehicle administration until the end of the experiment (C26 + sACVR/b), and 4) C26 TB-mice received continued sACVR administration throughout the experiment (C26 + sACVR/c), namely, in a prophylactic and continued manner.

**Figure 1:**
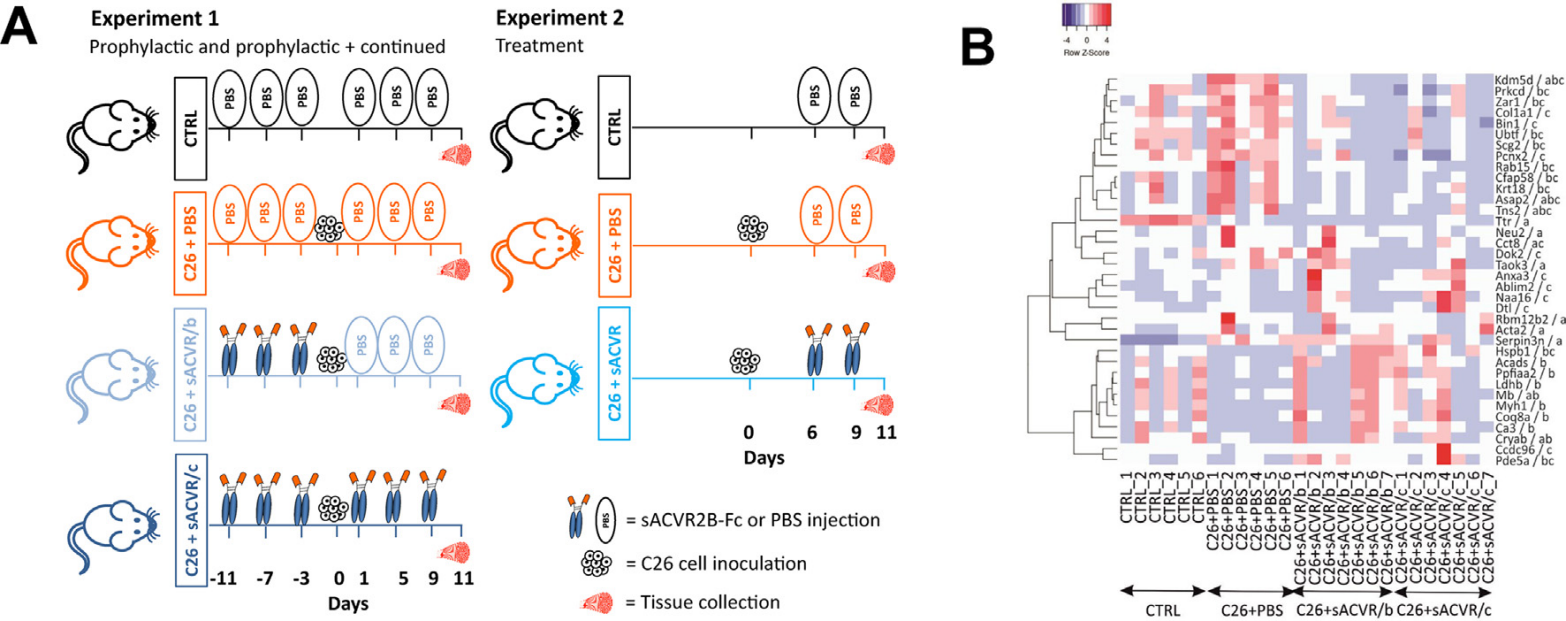
Overall study design (A). Experiment 1. Mice were inoculated with C26 cells on day 0, and sACVR (5 mg/kg) or PBS vehicle were administered on days −11, −7, −3, 1, 5, and 9. Muscles, liver, and blood were collected 11 days after the C26 cell inoculation. In experiment 2, sACVR (10 mg/kg) or PBS was injected only on days 6 and 9 after C26 cell inoculation. Heat map representation of differential proteome changes in 3 conditions showing proteins with the strongest response to cancer and anti-cachexia treatment in muscle in experiment 1 (B). For stringency, 3 conditions with changes in protein expression displaying only high fold change ratios are visualised (FC>|2.0|, FDR corrected p value < 0.05 and ≥ 2 unique peptides used for quantitation). C26 + PBS vs control (a), C26 + sACVR/b vs C26 + PBS (b) and C26 + sACVR/c vs C26 + PBS (c). An expression-based heat map was drawn by implementing average linkage and the Spearman rank correlation distance measure by using Heatmapper.

Experiment 2: mice were randomised into either 1) CTRL (+PBS), 2) C26 + PBS, or 3) sACVR injected after C26 tumour engraftment (C26 + sACVR). The experimental design is depicted in Figure 1A.

### 2.4 Experimental treatments

On day 0, mice were anaesthetised by ketamine and xylazine (Ketaminol® and Rompun®, respectively) and inoculated with 5 × 10^5^ C26 cells in 100 μl of PBS (TB-mice) or with an equal volume of vehicle (PBS) only (CTRL) into the intrascapular subcutis. Administration of 100 μl of sACVR diluted in PBS (5 mg/kg) or PBS vehicle was conducted intraperitoneally (i.p.). In experiment 1, sACVR (5 mg/kg) was injected twice per week, 3 times before C26 cell inoculation on days −11, −7, −3. For C26 + sACVR/c mice, sACVR was administered also after C26 cell inoculation, on days 1, 5, and 9. In the second experiment, sACVR (10 mg/kg) or PBS was injected as a treatment only on days 6 and 9 after tumour cell inoculation (Figure 1A).

### 2.5 The production of sACVR

The recombinant fusion protein was produced and purified as described earlier [12]. In summary, the ectodomain of human ACVR2B was fused with a human IgG1 Fc domain and expressed in Chinese hamster ovary cells grown in a suspension culture. The final injected protein is close to that originally generated by Lee and colleagues [13].

### 2.6 Tissue collection

On day 11 of the experiments, the mice were anaesthetised after an overnight *ad libitum* state by an i.p. injection of ketamine and xylazine (Ketaminol® and Rompun®, respectively), and euthanised by cardiac puncture followed by cervical dislocation. Tissue samples were rapidly excised, weighed, and snap-frozen in liquid nitrogen.

### 2.7 Muscle protein synthesis: *in vivo* surface sensing of translation

In experiment 2, muscle protein synthesis was analysed by using the surface sensing of translation (SUnSET) method [12,20,21]. Briefly, on day 11, after C26 cell inoculation, mice were injected i.p. with 0.04 μmol/g puromycin (Calbiochem, Darmstadt, Germany) dissolved in 200 μl of PBS. Tibialis anterior (TA) muscle was isolated, weighed, and snap-frozen in liquid nitrogen at 30 min after puromycin administration.

### 2.8 Sample preparation and quantitative label free proteomics analyses

Frozen muscle samples from experiment 1 (n = 7 per group) were thawed on ice; subsequently, they were homogenised in 2% SDS/0.1 M Tris pH 8/0.05 M DTT at room temperature by using tissue homogeniser. The homogenate was heated for 10 min at near 100 °C and cleared by centrifugation at 14 000 rpm (∼20 800 g) for 15 min. The protein concentration was determined by using the nanodrop technique; 10 μg of total protein was digested, using a modified FASP protocol [22]. To quantitatively assess the proteomics data, we used data independent acquisition-nano-LC-HDMS^E^ measurements as described in literature [23,24]. Database searches were conducted against UniProtKB/Swiss-Prot reviewed mouse (release 2017_16956 entries), with the ion accounting algorithm and by using the following parameters: peptide and fragment tolerance, automatic; maximum protein mass - 750 kDa; minimum fragment ions matches per protein ≥7; minimum fragment ions matches per peptide ≥3; minimum unique peptide matches per protein ≥1; digest enzyme-trypsin; missed cleavages allowed - 2; fixed modification - carbamidomethylation C; variable modifications - deamidation (N/Q) and oxidation of methionine (M); and false discovery rate, FDR <4%. The quality of the proteomic data was evaluated by PCA and by drawing a heatmap (Heatmapper; www.heatmapper.ca) [25] of all of the quantified proteins confirming the condition-specific clustering of the data according to the study groups.

### 2.9 Database mining of proteomics data

Relative protein quantification between samples using precursor ion intensities was performed with Progenesis QI™ Informatics for Proteomics software (Version 3.0, Nonlinear Dynamics/Waters). To identify differentially expressed proteins (DEPs), *P* values (by ANOVA) and fold change (FC) were set to <0.05 and > |1.3|, respectively. The quantification was performed based on ≥2 unique peptides. The filtered, false discovery rate (FDR < 0.05) corrected lists served as inputs into Ingenuity Pathway Analysis (IPA, Ingenuity Systems, Redwood City, CA; www.ingenuity.com).

### 2.10 Protein extraction and Western blotting

TA muscle (n = 7–8 per group in experiment 1 and n = 5–6 in experiment 2) was homogenised in ice-cold buffer with proper protease and phosphatase inhibitors [4,12] and centrifuged at 10 000 g for 10 min at +4 °C except for the SUnSET protein synthesis measurements, where samples were centrifuged at 500 g for 5 min. Total protein content was determined by using the bicinchoninic acid protein assay (Pierce, Thermo Scientific) with an automated KoneLab device (Thermo Scientific). Muscle homogenates containing 30 μg of protein were solubilised in Laemmli sample buffer and heated at 95 °C to denature proteins, separated by SDS-PAGE, and transferred to PVDF membrane, followed by overnight probing with primary antibodies at +4 °C. Muscle proteins were visualised by enhanced chemiluminescence using a ChemiDoc XRS or ChemiDoc MP device (Bio-Rad Laboratories) and quantified with Quantity One software version 4.6.3 (Bio-Rad Laboratories, Hercules, California, USA) or with Image Lab software (version 6.0; Bio-Rad Laboratories), respectively. In the case of puromycin-incorporated proteins, the intensity of the whole lane was quantified. Ponceau S staining and glyceraldehyde 3-phosphate dehydrogenase (GAPDH, ab9485: Abcam, Cambridge, USA) were used as loading controls, and the muscle protein level results were normalised to the mean of Ponceau S and GAPDH (experiment 1) or to the overall intensity on stain-free gels (experiment 2). To assess serum proteins, samples (n = 7–9 per group) were diluted 1:4, and the results were normalised to the total protein intensity on the membrane by using stain-free gels. Primary antibodies were against serpin family A member 3n (Serpina3n, AF4709: R&D Systems, Minneapolis, USA), p-STAT3 at Tyr^705^, total STAT3, p-ACC at Ser^79^, p-Akt at Ser^473^ and total Akt (Cell Signalling Technology, Danvers, USA), and fibrinogen (Abcam ab27913, Cambridge, UK).

### 2.11 RNA extraction, cDNA synthesis, and quantitative real-time PCR

Total RNA was extracted from gastrocnemius samples (n = 7–8 per group) by using QIAzol and purified with the RNeasy Universal Plus kit (Qiagen) according to manufacturer's instructions, resulting in high-quality RNA. RNA was reverse transcribed to complementary DNA (cDNA) with the iScriptTM Advanced cDNA Synthesis Kit (Bio-Rad Laboratories) according to kit instructions. Real-time qPCR was performed in triplicate according to standard procedures by using iQ SYBR Supermix (Bio-Rad Laboratories) or Maxima SYBR Green qPCR Master Mix (2x; Thermofisher Scientific) with CFX96 or CFX384 Touch™ Real-Time PCR Detection Systems and CFX Manager software (Bio-Rad Laboratories), respectively. Data analysis was conducted by using the efficiency corrected ΔΔCt method. Based on the lowest variation between and within the groups of the potential housekeeping genes (*Rn18S*, *Gapdh*, *36b4*, *Hprt* or *Tbp*), *36b4* or the mean of *36b4* and *Hprt* was chosen. Primers used are listed in Supplementary methods (Table S1).

### 2.12 NAD^+^ metabolism and mitochondrial *in situ* analyses

NAD^+^ and NADH were measured from gastrocnemius muscle (n = 6 per group) by using slightly modified conventional colorimetric enzymatic assays (see NADmed™ https://www.helsinki.fi/en/researchgroups/mitochondrial-medicine/nadmed). The activity of main cellular NAD^+^ consumers, poly (ADP-ribose) polymerases (PARPs) was determined from gastrocnemius muscle with the HT Colorimetric PARP/Apoptosis Assay Kit (Trevigen, Inc., Gaithersburg, MD, USA) according to manufacturer's instructions (n = 5–6 per group).

For mitochondrial activity *in situ* analysis, serial cross-sections (8 μm) from TA muscle were cut on a cryomicrotome (at −25 °C; experiment 1, encompassing mice from CTRL, C26 + PBS and C26 + sACVR/c groups). Mitochondrial activity was investigated by assessing the *in situ* activity of succinate dehydrogenase (SDH, complex II) and cytochrome c oxidase (COX, complex IV) from frozen TA muscle sections by using histochemistry. Cross-sectional images were digitally captured by using a Zeiss Axioplan 2 light microscope (Carl Zeiss AG, Germany) with a ProgRes C7 camera and ProgRes CapturePro 2.9.0.1 software (Jenoptik AG, Germany). Two fields-of-view per muscle cross-section were captured with a 5× magnification. A sample was excluded if cutting of the cross-section was unsuccessful (resulted in n = 5–7 per group). The COX and SDH *in situ* activities were analysed with Fiji/ImageJ (NIH, Bethesda, MD, USA) plugin Trainable Weka Segmentation [26]. Image resolution was reduced to 1/4 of the original because of the computational power required for analysis. Regions of interest were determined separately for each image and, after splitting channels, applied to green channel images to obtain optical density values of different fibre types. Optical density was assessed from all fibres and from high COX and high SDH fibres. Muscle distribution of mitochondria-rich, high SDH and COX fibres was calculated by using the ImageJ plugin Cell Counter and represented as percentage of high optical density fibres from all fibres.

### 2.13 Flow cytometry analysis to characterise splenomegaly in cancer

In the second experiment, the spleens of mice derived from 3 experimental groups (n = 6–8 per group) were harvested under sterile conditions, and single cell suspensions were prepared by gently pressing the minced spleens through 70 μm cell strainers. The total number of cells was calculated by counting the cell concentration by using a Beckton Dickinson Accuri C6 flow cytometer. Ten^6^ cells were then incubated with 0.4 mg/ml RNase A for 30' and stained with 18 μg/ml propidium iodide to distinguish between erythrocytes and nucleated cells and assess the percentage of proliferating cells.

### 2.14 Statistical analysis

Analyses (for proteomics analyses details, see above) were conducted with a general linear model ANOVA or Kruskall–Wallis test and then a two-tailed unpaired Student's t test or Mann–Whitney U test, respectively, when appropriate. Planned comparisons in all analyses were CTRL vs C26 + PBS and within TB-mice, either sACVR/c or sACVR/b (or sACVR in the second experiment) vs C26 + PBS and sACVR/c vs sACVR/b. When no clear effect of the sACVR administration was observed, the C26 cancer effect was analysed by pooling C26 groups vs CTRL to simplify the results and the figures. Pearson's correlation coefficient was used to assess the correlations. The level of significance was set at *P* < 0.05. Data were expressed as means ± SEM if not otherwise mentioned. Statistical analyses were performed with IBM SPSS Statistics version 24 for Windows (SPSS, Chicago, IL) and with GraphPad Prism 8.0.

## 3 Results

### 3.1 Characterisation of muscle proteome

Previously, we demonstrated that C26 cell inoculation resulted in muscle and fat wasting. Treating these TB-mice with sACVR before and after tumour formation prevented muscle loss and prolonged survival without affecting tumour size [4]. In this study, using label free proteomics, we identified 1232 proteins extracted from the murine muscles of these mice [4]. Notably, 876 proteins were quantified, followed by the exclusion of proteins considered contaminants, for example, blood haemoglobins or skin keratins (Table S2). The features of all groups were assessed by principal component analysis (PCA), which demonstrated a good group separation for TB and control mice (for a summary, see the panels in Figure S1). PCA also revealed 2 biological outliers that we excluded from subsequent analyses, resulting in n = 6–7 mice per group. We identified 119 DEPs in PBS-treated TB-mice. Among these, 53 proteins exhibited a higher expression level in TB-mice, and 66 were downregulated.

sACVR injection resulted in increased muscle size [4], accompanied by 126 DEPs (53 upregulated and 73 downregulated) with the prophylactic and continued (sACVR/c) administration, and by 117 DEPs (58 up- and 59 downregulated in their expression) in the prophylactic/discontinued (sACVR/b) administration, respectively, when compared to PBS-treated TB-mice. For the purpose of stringency, only the most altered proteins (FC > |2|) are presented in Figure 1, and the comparisons between the administration modalities are in Table S2.

### 3.2 Pathway analysis reveals decreased mitochondrial OXPHOS signature in TB-mice: a response attenuated in sACVR-treated mice

**Figure 2:**
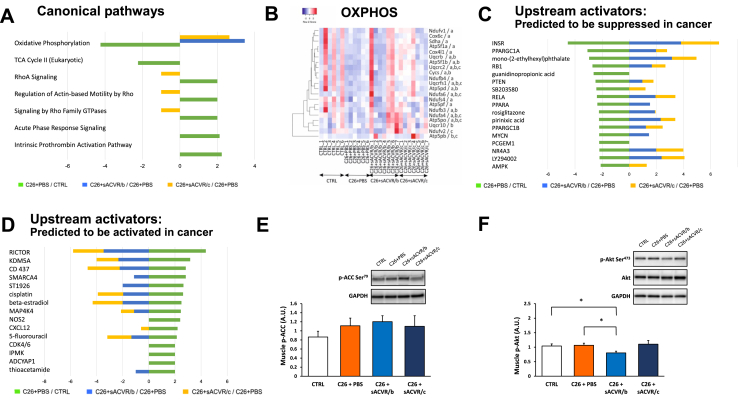
Ingenuity pathway analysis reveals pathways and upstream regulators affected in muscle by cancer and anti-cachexia treatment in experiment 1. Canonical pathways analysis (A). Pathways with significant z-scores (>|2|) in one of 3 comparisons are presented, and if available, z-scores of those pathways are also shown from other comparisons. Heat map representation (B) of OXPHOS proteins with largest differential changes in their expression, in 3 comparisons: (a) C26 + PBS vs control, (b) C26 + sACVR/b vs C26 + PBS, and (c) C26 + sACVR/c vs C26 + PBS. The criteria used for assessment were as follows: fold change, FC>|1.3|, FDR corrected p value < 0.05, and ≥2 unique peptides used for quantitation. An expression-based heat map using average linkage and the Spearman rank correlation distance measures. Upstream analysis pinpointing upstream regulators predicted to be suppressed (C) or activated (D) in C26 + PBS vs CTRL comparison (z-score >|2|) and if available, z-scores of those pathways are also shown from other comparisons. Phosphorylated ACC at ser^79^ as a proxy for AMPK activation (p-ACC; E) and p-Akt at Ser^473^ as a proxy for mTORC2 activation (F). ∗ = *P* < 0.05 using a two-tailed unpaired Student's t test.

Canonical pathway analysis of DEPs revealed a signature of decreased mitochondrial oxidative phosphorylation (OXPHOS) in the PBS-treated TB-mice (Figure 2A). Eighteen DEPs within this pathway included subunits of OXPHOS complex I (NADH: ubiquinone oxidoreductase), complex II (succinate dehydrogenase, SDH), complex III (ubiquinol-cytochrome c reductase), complex IV (cytochrome c oxidase, COX), and complex V (ATP synthase), suggesting a wide-spectrum downregulation of OXPHOS proteome (OXPHOSome; Figure 2B). This downregulation was in part (several proteins of CI-IV and ATP synthase subunits) prevented by sACVR/b (7 DEPs significantly upregulated compared to C26+PBS) and sACVR/c administration (12 DEPs upregulated vs C26+PBS), without an observable difference between sACVR/c and sACVR/b (Figure 2B). TB-mice also exhibited a consistent decrease in the expression of mitochondrial TCA cycle proteins (5 out of 5 quantified), which is in line with the reported decline in citrate synthase activity in the same TB-mice [4]. However, the blocking of ACVR ligands had no marked effect on the TCA cycle (Figure 2A).

**Figure 3:**
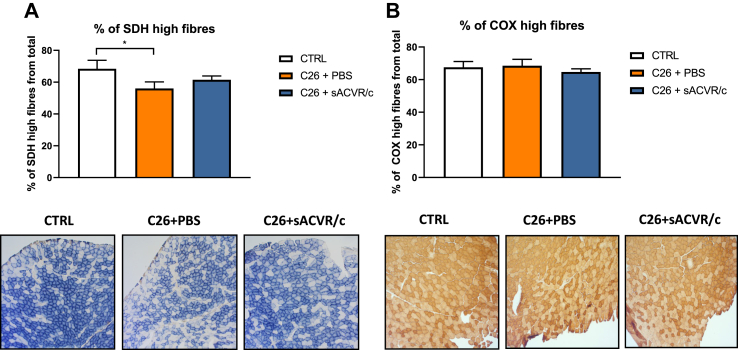
Muscle mitochondrial activity *in situ* is in part altered in cancer in experiment 1. Percentage of fibres with high SDH (A) or COX (B) activities. ∗ = *P* < 0.05 using a two-tailed unpaired Student's t test.

To further understand how the altered OXPHOSome signature affects mitochondrial metabolism, we determined *in situ* activities of complex II (SDH) and complex IV (COX) from the CTRL, C26+PBS, and C26+sACVR/c groups. In line with the OXPHOS, in some results showing complex II protein subunit downregulation, the percentage of muscle fibres endowed with high SDH activity (and thus mitochondria-rich fibres) significantly decreased in the PBS-treated TB-mice, and this decrease was not significant in mice administered by continued sACVR/c (Figure 3A). Different administration protocols did not induce changes in total SDH and COX activities (Figure S2) or in the percentage of muscle fibres with high COX activity (Figure 3B). In summary, decreased *in situ* SDH activity in TB-mice validated dysregulated OXPHOSome in cancer cachexia.

### 3.3 Decreased oxidative and anabolic signalling in TB-mice predicted by upstream factor analysis, the latter response rescued by sACVR

Unbiased upstream-regulator analysis suggested possible upstream regulators to explain the dysregulated mitochondrial signature (Figure 2C,D). More explicitly, it predicted that transcriptional regulatory proteins important for muscle mitochondrial biogenesis, OXPHOS and fatty acid oxidation, such as peroxisome proliferator-activated receptor gamma coactivator 1 alpha, and beta (PPARGC1A and PPARGC1B, namely, PGC-1α/β) [27], peroxisome proliferator-activated receptor-alpha (PPARA, i.e., PPARα) [28], and surprisingly, AMP-activated protein kinase (AMPK) [29] are also significantly suppressed (i.e. reduced activation, z-score < |2|) in TB-mice (Figure 2C). However, phosphorylated ACC at Serine^79^ status, often used as a proxy for AMPK activation, did not confirm suppressed AMPK-signalling in cachexia (Figure 2E). Among predicted upstream regulators, PGC-1α and PGC-1β were rescued by sACVR (Figure 2C).

Related to the decreased muscle mass in TB-mice and its treatment by continued sACVR administration [4], the reduced activation of insulin receptor (INSR) was predicted to be strongly linked to the C26+PBS vs CTRL scenario (a consistent decrease in all 22 DEPs in the dataset for the insulin receptor; Figure 2C). Particularly, the sACVR/c administration fully restored the activation of INSR in the same analysis (Figure 2C). The predicted upstream regulators activated in TB-mice are presented in Figure 2D. Connected to this pathway is the main muscle size regulating hub, mTORC1, which was shown to be suppressed in TB-mice and rescued by sACVR [4]. Surprisingly, Rictor, which refers to a mechanistic target of rapamycin complex 2 (mTORC2), was predicted by IPA to be strongly activated in PBS-treated TB-mice, and this response was prevented specifically in sACVR/c treated mice (Figure 2D). The prediction implies that activation of mTORC1 and mTORC2 is regulated in an inversed manner in cachexia and after the blocking of activin receptor ligands. Therefore, phosphorylated Akt at Serine^473^ status, a standard proxy for mTORC2 activation, was analysed. However, the result of this analysis did not confirm activated mTORC2-signalling in cachexia (Figure 2F).

### 3.4 Disturbed NAD^+^ homeostasis in TB-mice is rescued by the continued sACVR administration

**Figure 4:**
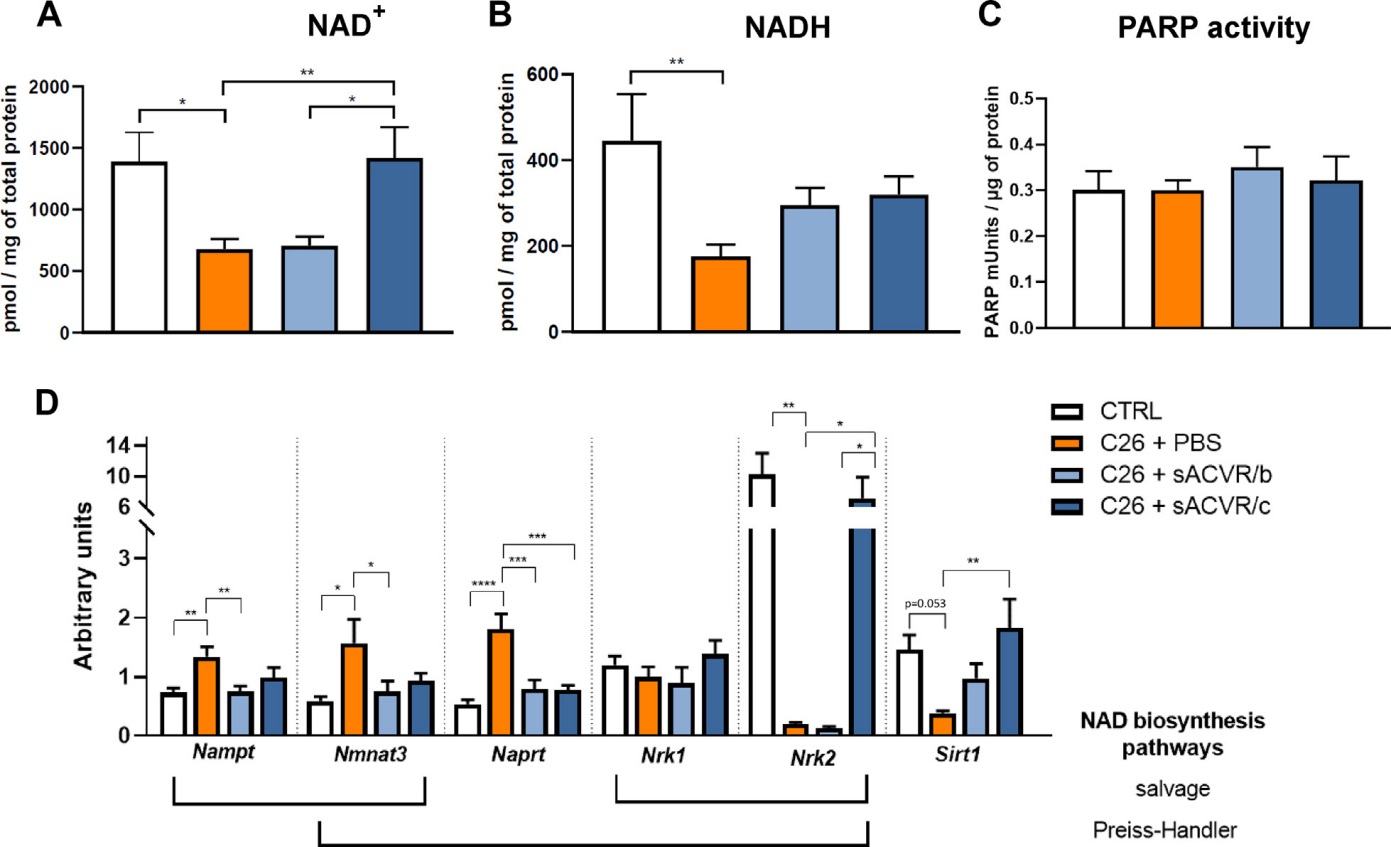
NAD^+^ and its regulators are affected in TB-mice and in part rescued by anti-cachexia treatment in experiment 1. Contents of NAD^+^ (A) and NADH (B), and PARP activity (C). Gene expression of the regulators of NAD^+^ biosynthesis (D). ∗ = *P* < 0.05, ∗∗ = *P* < 0.01, and ∗∗∗ = *P* < 0.001 when using a two-tailed unpaired Student's t test.

NAD^+^ metabolism is crucial for mitochondrial oxidative metabolism. Namely, the NAD^+^/NADH redox couple regulates the activity of the TCA cycle and OXPHOS, and the gene expression and activity of sirtuin 1 (*Sirt1*), the key inducer of PGC-1α activity [30]. Therefore, oxidised and reduced concentrations of NAD^+^ were assessed. We observed that the levels of both oxidised (NAD^+^) and reduced NAD (NADH) decreased in PBS-treated TB-mice (*P* < 0.05), and unlike sACVR/b, sACVR/c restored the depleted NAD^+^ levels; for NADH, this effect was not statistically significant (Figure 4A,B).

To uncover the mechanism behind NAD^+^ depletion, we next analysed NAD^+^ consumption and biosynthesis pathways in TB-mice. Given that the activity of the major cellular NAD^+^ consumers, PARPs, remained unchanged, the NAD^+^ depletion in TB-mice is unlikely to result from PARP-dependent NAD^+^ utilisation (Figure 4C). Notably, the expression analysis of the main muscle genes involved in NAD^+^ biosynthesis pathways (salvage and Preiss–Handler pathways) revealed a strongly diminished muscle isoform of nicotinamide riboside kinase (*Nrk*), *Nrk2*, in PBS-treated TB-mice, and sACVR/c completely rescued it (*P* < 0.05; Figure 4D). Similar to NAD^+^ levels, the sACVR/b administration modality demonstrated no effects on *Nrk2* expression (Figure 4D). By contrast, the expression of the ubiquitously expressed isoform, *Nrk1,* was similar in all groups (Figure 4D). The expression of other enzymes of the NAD^+^ biosynthesis pathway, namely, nicotinamide phosphoribosyltransferase (*Nampt*), nicotinamide adenylyl transferase 3 (*Nmnat3*), and nicotinate phosphoribosyltransferase (*Naprt*) increased in PBS-treated TB-mice, a feature that was normalised by sACVR (Figure 4D).

The *de novo* NAD^+^ biosynthesis pathway is considered to operate at a low level in skeletal muscle and thus may have low physiological significance. Nevertheless, we observed increased tryptophan 2,3-dioxygenase (*Tdo)* and quinolinate phosphoribosyltransferase (*Qprt)* expression in the PBS-treated TB-mice, and these responses tended to be preserved in the sACVR-treated condition (Figure S3). Downstream to NAD^+^, TB-mice demonstrated downregulation of NAD^+^ dependent *Sirt1* (*P* = 0.053, Figure 4D), which acts as an important protein deacetylase, protecting cells from metabolic stress. In line with the NAD^+^ and *Nrk2* results, *Sirt1* expression was rescued by sACVR/c, and less so by sACVR/b (Figure 4D). Overall, our data suggest that cancer cachexia is characterised by muscle NAD^+^ deficiency which can be restored by sACVR when administered before and after tumour injection.

### 3.5 Therapeutic ACVR ligand blockade restores muscle mass, protein synthesis, NAD⁺ levels and *Nrk2*

**Figure 5:**
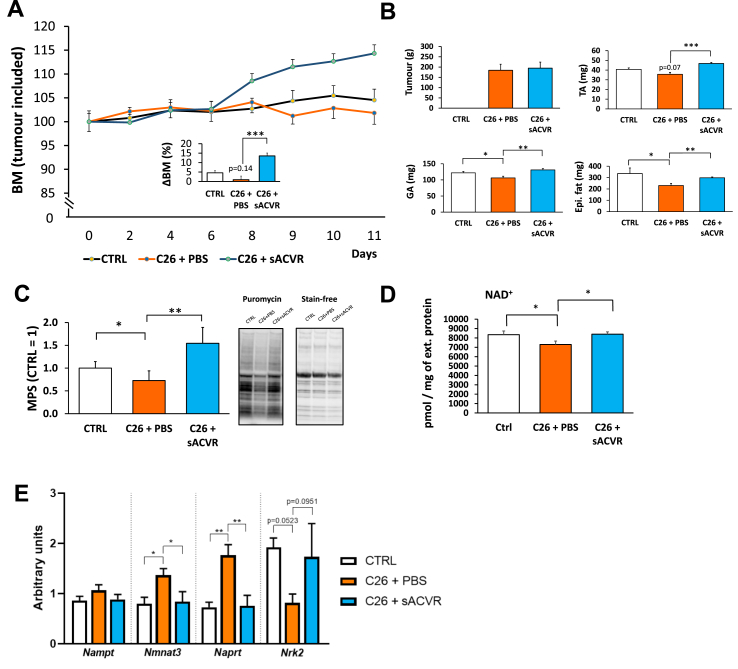
Anti-cachexia treatment after tumour inoculation (experiment 2) reveals increased body mass, muscle masses, and muscle protein synthesis and repleted NAD^+^. Changes in body mass (BM; A), tissue masses (B), muscle protein synthesis (C), contents of NAD^+^ (D), and gene expression of the regulators of NAD^+^ biosynthesis (E) in control mice and in mice treated with sACVR or PBS. Epi. fat = epididymal fat pad. ∗ = *P* < 0.05, ∗∗ = *P* < 0.01, and ∗∗∗ = *P* < 0.001 when using a two-tailed unpaired Student's t test.

In addition to the prophylactic and/or prophylactic and continued ACVR blocking, we next conducted a more translatable experiment in which the sACVR administration was initiated after tumour formation. We observed that sACVR treatment increased BM (Figure 5A) and rescued the decrease in muscle mass (Figure 5B) and protein synthesis in TB-mice (Figure 5C) without affecting the tumour growth (Figure 5B). We also replicated the findings of experiment 1 and showed that muscle NAD^+^ is depleted in C26 TB-mice even when cachexia is very mild (Figure 5D), and this was accompanied by decreased *Nrk2* expression (P = 0.052; Figure 5E). Therapeutic sACVR treatment restored levels of NAD^+^ (Figure 5D), and a similar trend was observed for *Nrk2* (P = 0.095; Figure 5E); both results are in line with the results of sACVR/c administration. To support the findings from experiment 1, we also replicated the results of *Nmnat3* and *Naprt* gene expression but not of *Nampt* (Figure 5E). However, the expression of *Nrk1* and *Sirt1* was very low and thus not quantified. In summary, these data validated the tumour-effects observed in a previous experiment and showed that muscle NAD^+^ deficiency can be restored by exposure to therapeutic sACVR.

### 3.6 Pathway and upstream analyses reveal acute phase response in TB-mice

**Figure 6:**
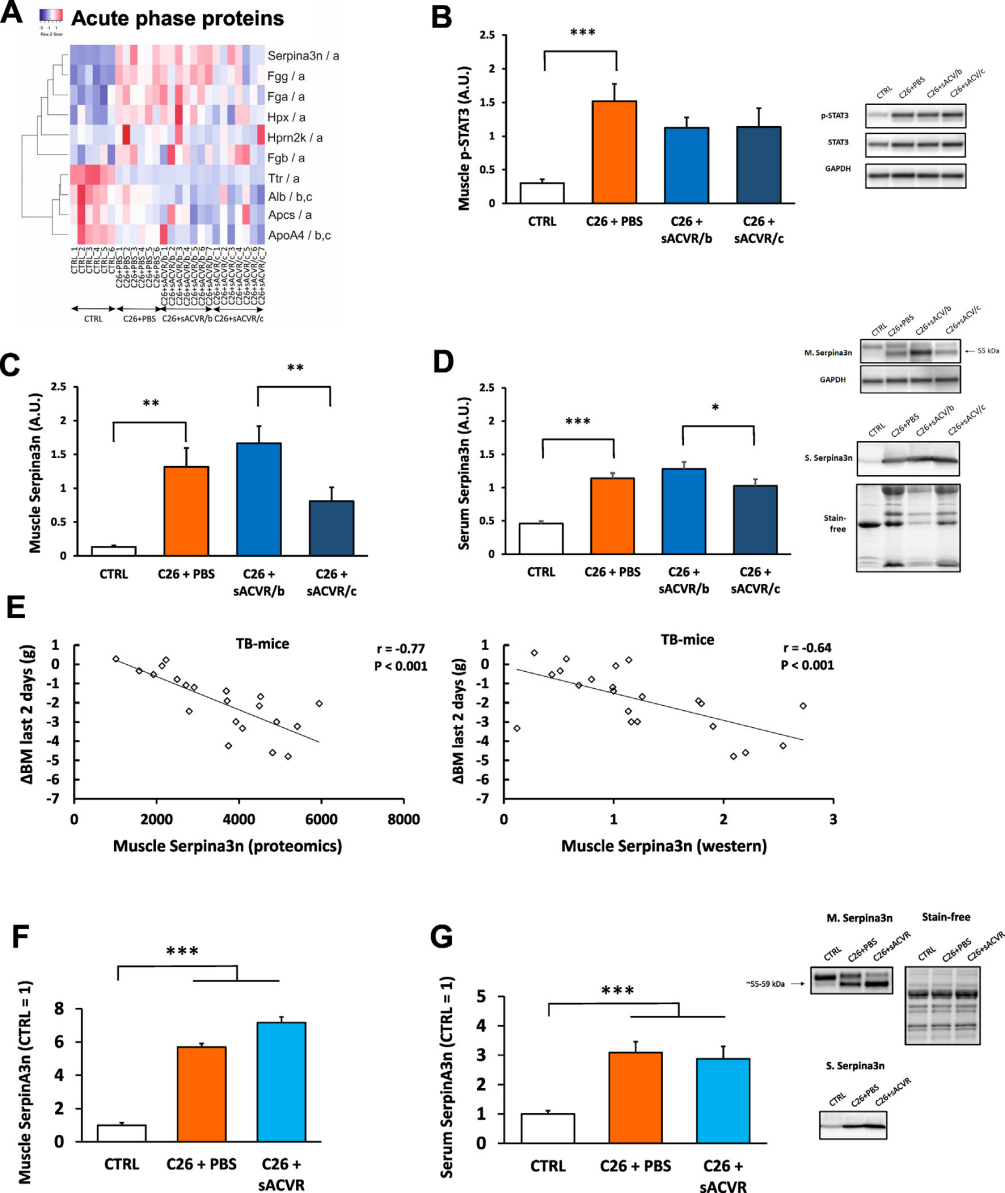
APR is activated in TB-mice independent of anti-cachexia treatment. Heat map representation of differential proteome changes of APPs in experiment 1. The analyses in 3 conditions with the highest fold change ratio are shown (FC>|1.3|, FDR corrected *P* value < 0.05 and ≥ 2 unique peptides used for quantitation), namely: (a) C26 + PBS vs control, (b) C26 + sACVR/b vs C26 + PBS, and (c) C26 + sACVR/c vs C26 + PBS. An expression-based heat map is using average linkage and the Spearman rank correlation distance measures (A). Phosphorylated STAT3 (p-STAT3; B) and Serpina3n quantifications in muscle (C) and in serum (D). Correlation analysis of Serpina3n in the muscle, quantified either by label free proteomics or with Western blotting and cachexia (a decrease in the body mass, BM; E). Serpina3n in muscle (F) and in serum (G) in mice treated with sACVR/PBS after tumour cell inoculation in experiment 2. ∗ = *P* < 0.05, ∗∗ = *P* < 0.01, and ∗∗∗ = *P* < 0.001 when using a two-tailed unpaired Student's t test.

Pathway analysis predicted involvement and upregulation of acute phase proteins (APPs) reflecting acute phase response (APR) in cancer (Figure 2, Figure 6A). Corresponding upstream analysis indicated that activated STAT3 (*P* = 2.17∗10^−7^, Z-score 1.45, not shown) may represent a regulator for this response, as shown by Bonetto et al. [31]. Subsequently, Western blotting analysis confirmed increased phosphorylation of STAT3 in TB-mice (Figure 6B). The STAT3 signalling pathway is downstream to IL-6 and MCP-1, both being elevated in the serum of TB-mice [4], which may connect mitochondrial dysfunction to muscle cachexia [2].

### 3.7 APP Serpina3n, the most altered protein in TB-mice, is associated with the loss of BM but not affected by the anti-cachexia treatment

Among identified DEPs within the APR pathway, Serpina3n (human orthologue of Serpina3, also known as Alpha-1-antichymotrypsin) represented the most strongly upregulated protein (Figure 1, Figure 6A), whose increased expression was validated in cachectic muscle by Western blotting (Figure 6C). Elevated Serpina3n levels were also observed in the serum of TB-mice (Figure 6D). The increase in Serpina3n protein was probably due to the transcriptional regulation of the gene because the level of muscle mRNA expression of *Serpina3n* increased in TB-mice (Figure S4), and it correlated well with the Serpina3n protein level (r = 0.67, *P* = 0.001). Serpina3n was higher in the sACVR/b group than in the sACVR/c (*P* < 0.001) group, without an observable difference when compared with PBS-treated TB-mice (Figure 6A,C). A similar effect was traced on the qPCR analysis (Figure S4), demonstrating that Serpina3n protein and mRNA levels are most upregulated in a group of mice with the strongest decrease in BM during the last days before the tissue collection [4].

These results and the earlier evidence [32] suggest that Serpina3n may represent a biomarker related to changes in muscle/BM. Indeed, muscle Serpina3n protein strongly correlated with the loss of BM at 9–11 days after tumour cell engraftment (r = −0.77, *P* < 0.001) in TB-mice (Figure 5E). A similar observation was made at the mRNA level (r = −0.84, *P* < 0.001, Figure S5A). At this time point, the loss of BM predicted survival (Cox regression: B = 0.90, *P* < 0.001) in another group of mice [4]. The proteomics result was validated by Western blotting in TB-mice (r = −0.64, *P* < 0.001, Figure 6E). In serum (r = −0.69, *P* < 0.001) and in the liver (r = −0.82, *P* < 0.001), Serpina3n levels also correlated with BM loss at the same time point (Figure S5A). These results demonstrate that high Serpina3n expression (at the mRNA and protein level) is related to a significant loss of BM in TB-mice.

The gene expression of other APPs, namely, *Saa1* and *Fibrinogen G* (Figure S4), as well as serum Fibrinogen, were elevated in TB-mice (Figure S5B). Unlike Serpina3n, serum Fibrinogen correlated with BM loss only when all mice were included (r = −0.58, *P* < 0.001), but not in TB-mice (r = −0.32, *P* = 0.154; Figure S5B). In skeletal muscle, APP components were not affected by sACVR (Figure 6A).

We subsequently conducted a correlation analysis including all proteins identified in muscle proteomics. Among those, Serpina3n clearly correlated most strongly with the loss of BM at 9–11 days after tumour cell engraftment followed by fibrinogen G (data not shown).

### 3.8 Cachexia-associated Serpina3n reflects the inflammatory state

In the second experiment, we replicated the findings of increased muscle (Figure 6F) and serum (Figure 6G) Serpina3n independently of sACVR treatment, even though sACVR rescued cachexia and muscle protein synthesis. Furthermore, muscle Serpina3n correlated with BM loss in TB-mice only when sACVR-treated mice (with strongly increased BM, muscle mass, and muscle protein synthesis) were excluded from the analysis (Figure S6). To further analyse the impact of inflammation in TB-mice and whether Serpina3n reflects on it, we analysed the spleen size and its morphology. Spleen size increased in TB-mice, and this was not affected by the sACVR treatment (Figure S7). To investigate the C26-induced spleen enlargement, namely, splenomegaly in more detail, a flow cytometry analysis was conducted, which demonstrated the process that stemmed from both increased erythrocyte engulfment and white blood cell proliferation (Figure S7). Spleen mass, as well as serum IL-6, correlated with muscle Serpina3n (Figure S6).

## 4 Discussion

In this study we characterised the muscle proteome of TB-mice with or without anti-cachexia treatment. Our proteomics results demonstrated a reduction in the expression of proteins implicated in OXPHOSome, a finding further corroborated by measurements pinpointing dysregulated NAD^+^ metabolism and alterations in NAD^+^ biosynthesis including downregulation of *Nrk2* in cancer cachexia. These changes were rescued by sACVR when administered to TB-mice. In subsequent experiments we demonstrated that sACVR treatment that commenced after tumour formation prevented a cancer-induced decrease in muscle protein synthesis and NAD^+^ but not splenomegaly. Finally, we established evidence for acute phase protein Serpina3n as the most consistent predictor of untreated cachexia in murine muscle proteome but not as a generic marker of alterations in muscle mass.

The decrease in the OXPHOSome, the lowered expression of components in the TCA-pathway, and the low percentage of muscle fibres with high SDH activity in TB-mice support the published observations of mitochondrial dysfunction in cancer cachexia demonstrated by using proteomics [33], transcriptomics [34], and mitochondrial oxidative enzyme activity analyses [4,35]. However, based on Western blot assay, the protein content of few OXPHOS proteins has also been reported to remain relatively unaltered in cachectic TA muscle [4]. In cancer cachexia, muscle mitochondrial dysfunction may occur in part through cytokine-signalling such as IL-6 – STAT3 axis [2], confirmed by this study; alternatively or complementary to this, reduced physical activity observed in these TB-mice [4] may contribute to the process of dysregulated mitochondria [2] that per se may be sufficient to induce muscle atrophy [36]. Utilising proteomics, we demonstrated that blocking ACVR ligands attenuated these changes in OXPHOSome, but no significant differences were observed in the response between the administration modalities. Previously, blocking ACVR ligands has either had no effect [15] or even diminished mitochondrial oxidative capacity [14,37,38], and decreased OXPHOS-related protein expression in muscle [39] while inducing fast and glycolytic transformation [40]. However, blocking ACVR ligands has also resulted in positive outcomes in various disease, injury, and ageing models [[4], [5], [6],^15^,[40], [41], [42]]. Our results imply that blocking ACVR ligands can have context-dependent effects on muscle oxidative metabolism. Whether the rescue of OXPHOSome is linked to improved survival [4] requires further research.

As predicted by the upstream-regulator analysis, the diminished activities of PGC-1 α/β and PPARα may contribute to the global OXPHOSome alterations in cachectic muscle. Their deletion causes impairment of mitochondrial OXPHOS transcription and oxidative metabolism especially in stress conditions in mice [27,28]. We have observed a decrease in muscle PGC-1α in mice inoculated with C26 cells [35], and a similar tendency was observed in these mice, in TA muscle [4]. Moreover, dysregulated NAD^+^/sirtuin metabolism may contribute to the altered OXPHOSome signature in cachectic muscle. Notably, both oxidised and reduced NAD^+^ levels were significantly lowered in untreated TB-mice. In addition, a moderate decrease in the key regulator of muscle mitochondrial oxidative metabolism and stress defence, NAD^+^ -dependent *Sirt1,* was observed in cancer cachexia, as was shown in TB-mice in relation to the severity of cancer cachexia [43]. The continued and post-tumour ACVR treatment replenished NAD^+^ levels, and continued administration also normalised the expression of *Sirt1* similar to the predicted activities of its downstream targets PGC-1α/β. NAD^+^ depletion and a subsequent disturbance in cellular redox status can alter the function of various pathways such as mitochondrial energy production, cellular signalling, chromatin modulation, DNA repair, ROS defence, inflammation and immune responses, and circadian rhythm [44]. Previously, we demonstrated that sACVR altered redox balance and offered antioxidant protection in the muscles of these TB-mice by restoring the decreased levels of reduced glutathione (GSH) [17]. Therefore, sACVR can enhance the function of a wide range of cellular processes. In line with our results, several animal studies have demonstrated that NAD^+^ boosting is a promising therapy option to treat diseases with multifactorial pathology, for example, muscle dystrophy and myopathy, obesity, diabetes, fatty liver, and cardiac diseases [44]. Further studies should investigate whether NAD^+^ boosters offer an advantage in cancer cachexia. Overall, our work establishes a novel finding of NAD^+^ depletion in cancer cachexia, which can be restored by sACVR treatment in TB-mice.

This study also revealed that NAD⁺ depletion in cachectic muscle is associated with the altered expression of genes involved in NAD⁺ biosynthesis pathways but not with enzymatic activities of NAD⁺ consumers. Namely, the decline in NAD^+^ levels probably did not originate from the increased activity of PARPs, the major NAD^+^ consumers in cells [45], because the total PARP activity was unaltered in TB-mice. The analysis of NAD^+^ biosynthesis proposes *Nrk2 (Nmrk2),* an enzyme involved in the salvage pathway, is potentially associated with low muscle NAD^+^ content in cancer cachexia. By contrast, the gene expression of several other enzymes in the NAD^+^ biosynthesis pathway was elevated in PBS-treated TB-mice. These findings are puzzling because the deletion of *Nrk2* has not been demonstrated to affect muscle NAD^+^ levels because Nrk2 is redundant with other proteins such as Nrk1 in the NAD^+^ salvage pathway [46,47]. Thus, the question raised is whether muscle *Nrk2* downregulation results in a depletion of NAD^+^ levels in cancer cachexia or whether *Nrk2* downregulation is related to its other functions such as modulation of muscle mass. Another question raised here is whether Nrk2 downregulation is related to its other functions such as regulation of muscle fibre size, as reported in the literature [47]. *Nrk2* deficiency has also been shown to increase muscle expression of cytokine *Il-15,* a potent regulator of muscle growth. We, however, did not observe major changes in muscle *Il-15* (Figure S3). Overall, our studies revealed downregulation of *Nrk2* in cancer cachexia and its restoration by sACVR. Nevertheless, further research should investigate the regulation of *Nrk2* via TGF-β superfamily cytokines and the role of *Nrk2* in the maintenance of muscular NAD⁺ pool during pathological conditions such as cancer cachexia. Further research should also examine whether the myostatin/activin blockage repletes NAD^+^ and Nrk2 deficiencies via direct action or by interfering with cachexia-altered signalling events.

APR is an early response in cancer belonging to the innate immune system characterised by increased expression of positive APPs [48]. The APR protein Serpina3n belongs to a family of serine protease inhibitors. Serpina3n contains a signal peptide and is secreted into C2C12 muscle cell media after atrophic stimuli (i.e. glucocorticoids) [32], and in humans, it may be exported to blood through small vesicles [49], suggesting that Serpina3 (or Serpina3n) may indeed be a myokine. As demonstrated in proteomics experiments, Serpina3n was the most strongly associated protein with the loss of BM. Similarly, elevated Serpina3n levels in plasma and liver strongly correlated with cachexia. This occurred at a time point in which cachexia was predictive for a large variation in survival [4]. Our results are in accordance with the results of human studies connecting increased APR with compromised survival in cancer cachexia [50,51]. Although APR may assist in limiting host infection and tissue injury, its prolonged action may further contribute to the severity of cachexia [51].

The research on C26-induced cachexia has described an elevated expression of Serpina3n at mRNA [34] and protein [32] levels and that Serpina3n is associated with muscle mass [32]. Our findings on Serpina3n upregulation support these studies and also show that of the whole muscle proteome, Serpina3n was the best predictor of cachexia at a time point when cachexia predicts survival [32]. This finding, however, does not necessarily imply that the protein is causally related to cachexia or survival. To understand whether Serpina3n is influenced by changes in muscle protein synthesis and mass, we modulated these factors by treating TB-mice with sACVR after tumour inoculation and thus only a few days before the mice were euthanised. This demonstrated that muscle Serpina3n does not seem to represent a *bona fide* marker of alterations in muscle mass because i) muscle Serpina3n also increased in the sACVR-treated mice with a rescue of muscle mass, and ii) muscle Serpina3n correlated with the loss of BM only when the sACVR-treated mice were excluded from the analysis. Instead of being a direct regulator of muscle mass, increased Serpina3n may have various other effects. For instance, it correlated with spleen mass and serum IL-6, another strongly altered responses in cancer, showing that Serpina3n may reflect the inflammatory state in various tissues. The C26-induced splenomegaly originated from erythrocyte engulfment and white blood cell proliferation, both of which remained unaffected by sACVR administered only after tumour cell injection. This finding does not support our previous findings of splenomegaly mitigation after sACVR administration also before tumour formation [4]. In addition, increased muscle Serpina3n may also have localised effects in muscle extracellular space such as evoking decreased activity of proteases and thus protecting muscle from acute injury and muscle dystrophy [52]. This putative, protective effect of Serpina3n is also of interest in C26-TB mice, in which membrane abnormalities further accentuate muscle wasting [53]. Surprisingly, Serpina3n also correlated with muscle *Nrk2* (r = −0.72 and −0.74 in all mice and in TB-mice, respectively, *P* < 0.001, Figure S8) suggesting a possible connection of APR to *Nrk2* downregulation.

In conclusion, our results reveal disturbed OXPHOSome and NAD^+^ metabolism as a molecular determinant of cancer cachexia, which were rescued upon ACVR ligand blockade, depending on the used administration modality. We also present evidence implicating Serpina3n as a predictor of untreated cachexia but not as a generic marker of alterations in muscle mass.

## Author contribution

**J.J.H.**: Conceptualisation; Formal analysis; Funding acquisition; Investigation; Project administration; Resources; Visualisation; Writing - original draft & editing. **F.P.** Conceptualisation; Formal analysis; Funding acquisition; Investigation; Validation; Writing - review & editing. **N.P.** Formal analysis; Visualisation; Writing - review & editing. **T.N.** Conceptualisation; Investigation; Writing - review & editing. **J.H.** Investigation. **L.E.** Methodology. **J.H.L.** Formal analysis. **B.R.** Investigation. **R.S.** Formal analysis, Methodology. **M.B**. Methodology. **O.R.** Methodology; Resources. **E.P.** Formal analysis; Funding acquisition; Visualisation; Writing - review & editing. **M.L.** Data Curation, Formal analysis; Visualisation; Writing - review & editing

## Funding

This work was supported by the 10.13039/501100002341Academy of Finland (grant No. 275922 to JJH and 286359 to EP) and 10.13039/501100006383Cancer Society of Finland to JJH. The research leading to these results has also received funding from 10.13039/501100005010AIRC under IG 2018 - ID. 21963 project (FP).

## Appendix A Supplementary data

Multimedia component 1 **Supplementary Table S1**. Primer information for qPCR analyses. **Supplementary Table S2**. Proteomics: all quantified proteomics results. Presented as a separate Excel-file. **Figure S1**. Principal component analyses (PCA) demonstrating good separation between non-treated CTRL and TB-mice and 2 biological outliers, which were subsequently removed from other analyses. **Figure S2**. Total SDH and COX activities were unchanged in TB-mice. **Figure S3**. De novo pathway genes and Il-15 mRNA. ∗ = *P* < 0.05, ∗∗ = *P* < 0.01. **Figure S4**. Muscle APP mRNA’s from the first experiment. *Fgg* = fibrinogen g and *Saa* = Serum amyloid A. ∗ = *P* < 0.05, ∗∗ = *P* < 0.01. **Figure S5**. Correlation analysis of *Serpina3n* in muscle, liver, and serum, quantified from all mice or from TB-mice only in experiment 1. **Figure S6**. Correlation analysis between muscle Serpina3n and body mass (BM), spleen mass, or Serum IL-6 in experiment 2. ∗∗∗ = *P* < 0.001. **Figure S7**. Spleen mass, cell number, and differentiation between different spleen cells. **Figure S8**. Correlation analysis between muscle Serpina3n and *Nrk2* in experiment 1. Multimedia component 1
