# Supplementary material for: Muscle NAD+ depletion and Serpina3n as molecular determinants of murine cancer cachexia—the effects of blocking myostatin and activins
Source: Mol Metab. 2020 Jun 26;41:101046. doi: 10.1016/j.molmet.2020.101046 (PMC7364159; doi:10.1016/j.molmet.2020.101046)

Supporting information

Supplementary Table S1. Primer information for qPCR analyses.

Primer	Forward (5' - 3')	Reverse (5' - 3')
36b4	GGCCCTGCACTCTCGCTTTC	TGCCAGGACGCGCTTGT
Hprt1	GAGGAGTCCTGTTGATGTTGCCAG	GGCTGGCCTATAGGCTCATAGTGC
Il-15	TCCACATCTAACAGCTCAGAGAG	AGCAAGGACCATGAAGAGGC
Nampt2	GCGAGCGAGCGGTGACT	CTGCGAGCAAGGAGAAAAATG
Naprt	AGCCTCGTAGCCACCAATG	CTGCAATCAAGCGTAGCCG
Nmnat3	TCACCCGTCAATGACAGCTAT	CACCCGAATCCAGTCAGATGT
Nrk1	CCCAACTGCAGCGTCATATC	CCTTGAGCACTTTCCAAGGC
Nrk2	CACCTCAGGACCAGTCACCT	CTGTTGGTCAGGGTGGTCTT
Qprt	CCGGGCCTCAATTTTGCATC	GGTGTTAAGAGCCACCCGTT
Sirt1	GTCTCCTGTGGGATTCCTGA	ACACAGAGACGGCTGGAACT
Tdo	TGCTCAAGGTGATAGCTCGGA	AGGAGCTTGAAGATGACCACCA
Serpina3n	Bio-Rad Assay ID	qMmuCID0024737
Fgg	Bio-Rad Assay ID	qMmuCID0015216
Saa1	Bio-Rad Assay ID	qMmuCID0007991

Supplementary Table S2. Proteomics: all quantified proteomics results. As a separate file: TableS2.xlsx

Figure S1. Principal component analysis (PCA) shows clear separation between non-treated CTRL and TB-mice and 2 biological outliers in CTRL and C26 + PBS mice that were further excluded from the subsequent analyses resulting in n=6-7 mice per group in proteomics analysis.

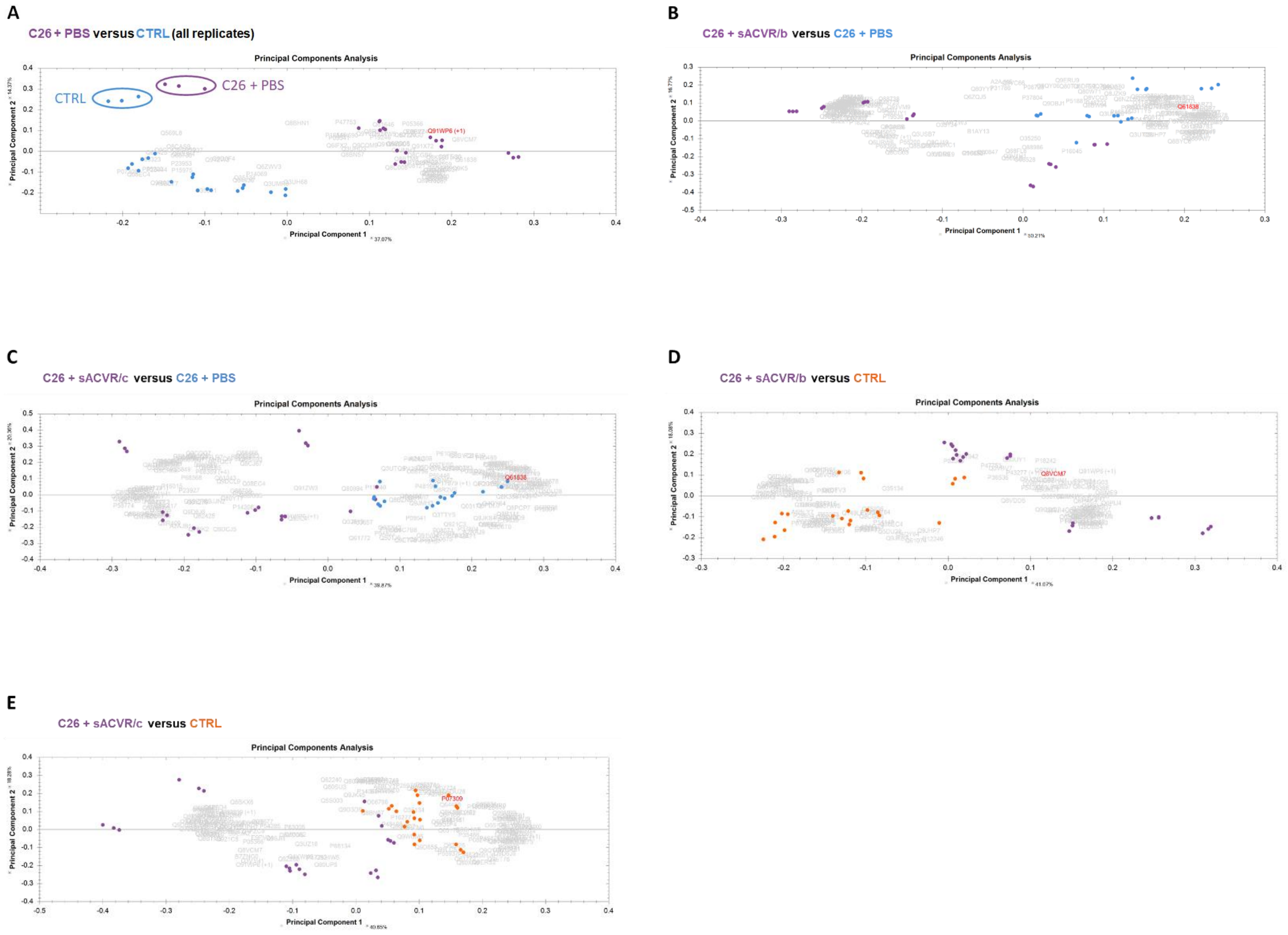

Figure S2. Total SDH and COX activities were unchanged in TB-mice.

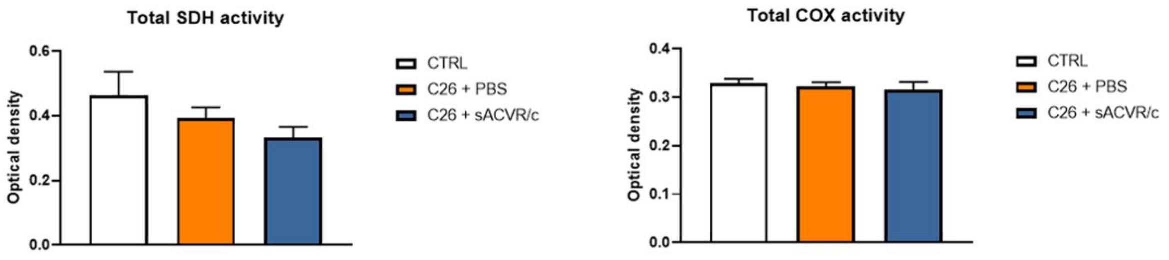

Figure S3. De novo pathway genes and Il-15 mRNA. * = $P < 0.05$, ** = $P < 0.01$ using a two-tailed unpaired Student's t-test.

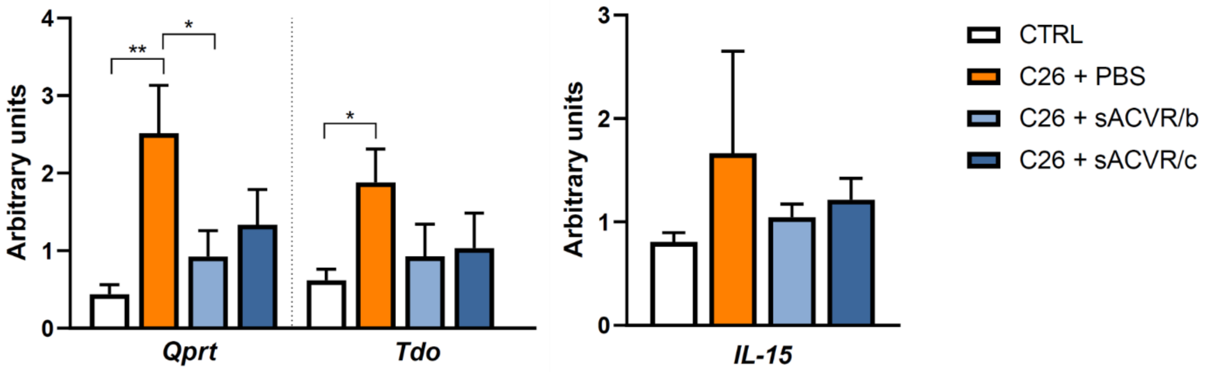

Figure S4. Muscle APP mRNA´s from the first experiment. *Fgg* = fibrinogen g and *Saa* = Serum amyloid A. * = $P < 0.05$, ** = $P < 0.01$ using Mann-Whitney U test.

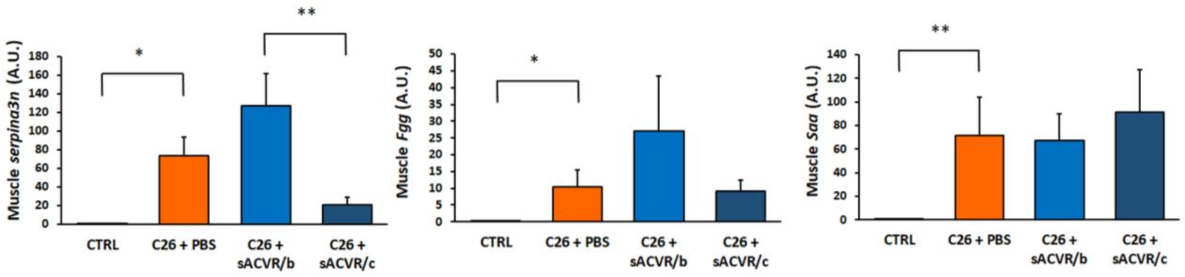

Figure S5. Correlation analysis of *Serpina3n* in muscle, liver and serum, quantified from all mice or from TB-mice only in experiment 1.

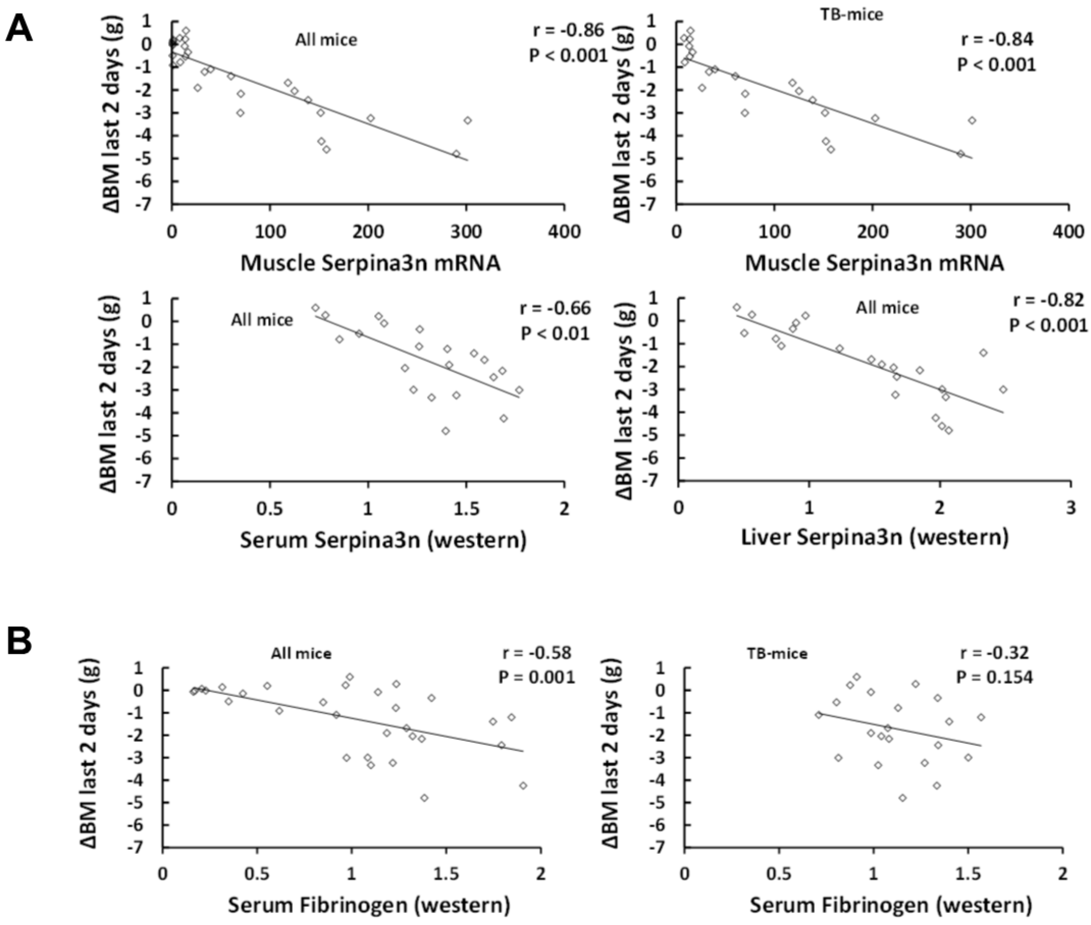

Figure S6. Correlation analysis between muscle Serpina3n and body mass (BM), spleen mass or Serum IL-6 in experiment 2. *** = $P < 0.001$ using a two-tailed unpaired Student’s t-test.

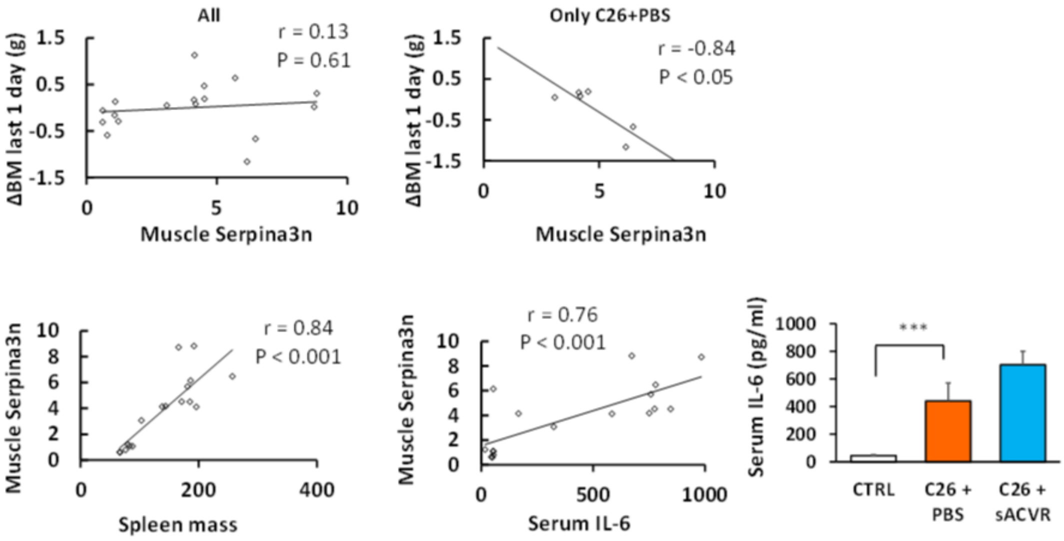

Figure S7. Spleen mass, cell number and differentiation between different spleen cells. WPC = White blood cells. C = CTRL, P = C26 + PBS and A = C26 + sACVR. Ext. = extracted. * = P < 0.05, ** = P < 0.01, *** = P < 0.001 using a two-tailed unpaired Student's t-test.

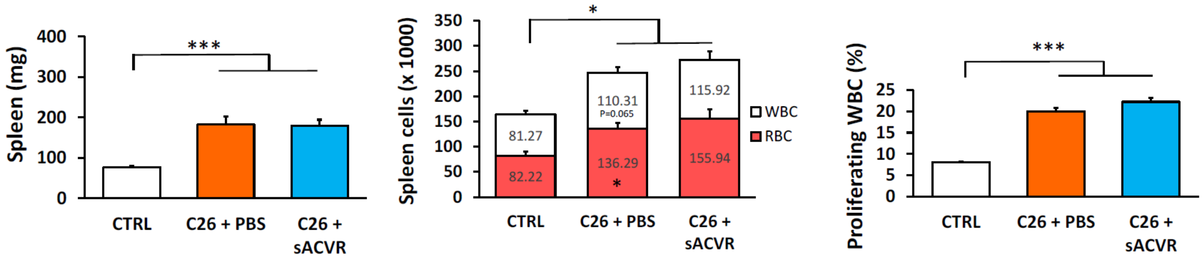

Figure S8. Correlation analysis in muscle between Serpina3n and *Nrk2* in experiment 1.

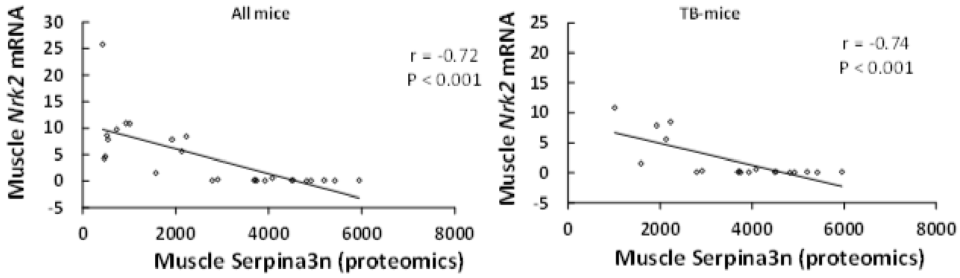

Supplement: Multimedia component 2 [file mmc2.pdf]
